# Supplementary material for: Preeclampsia and its determinants in Ethiopia: A systematic review and meta-analysis
Source: PLoS One. 2023 Nov 14;18(11):e0287038. doi: 10.1371/journal.pone.0287038 (PMC10645334; doi:10.1371/journal.pone.0287038)
Supplement: S1 Table — (DOCX) [file pone.0287038.s002.docx]

Searching Pharases

1. ("pre-eclampsia"[MeSH Terms] OR "pre-eclampsia"[All Fields] OR ("pre"[All Fields] AND "eclampsia"[All Fields]) OR "pre eclampsia"[All Fields]) AND determinants[All Fields] AND ("ethiopia"[MeSH Terms] OR "ethiopia"[All Fields])= 908
2. Magnitude[All Fields] AND ("pre-eclampsia"[MeSH Terms] OR "pre-eclampsia"[All Fields] OR ("pre"[All Fields] AND "eclampsia"[All Fields]) OR "pre eclampsia"[All Fields]) AND determinants[All Fields] AND ("ethiopia"[MeSH Terms] OR "ethiopia"[All Fields])=289
3. Magnitude[All Fields] AND ("pre-eclampsia"[MeSH Terms] OR "pre-eclampsia"[All Fields] OR ("pre"[All Fields] AND "eclampsia"[All Fields]) OR "pre eclampsia"[All Fields]) AND associated[All Fields] AND factors[All Fields] AND ("ethiopia"[MeSH Terms] OR "ethiopia"[All Fields])=427
4. Magnitude[All Fields] AND ("hypertension"[MeSH Terms] OR "hypertension"[All Fields] OR ("hypertensive"[All Fields] AND "disorder"[All Fields]) OR "hypertensive disorder"[All Fields]) AND ("pregnancy"[MeSH Terms] OR "pregnancy"[All Fields]) AND Determinant[All Fields] AND ("ethiopia"[MeSH Terms] OR "ethiopia"[All Fields])=282
5. ("epidemiology"[Subheading] OR "epidemiology"[All Fields] OR "prevalence"[All Fields] OR "prevalence"[MeSH Terms]) AND ("pre-eclampsia"[MeSH Terms] OR "pre-eclampsia"[All Fields] OR "preeclampsia"[All Fields]) AND Determinant[All Fields] AND ("ethiopia"[MeSH Terms] OR "ethiopia"[All Fields])=340

Google scholar

1. ("pre-eclampsia"[MeSH Terms] OR "pre-eclampsia"[All Fields] OR ("pre"[All Fields] AND "eclampsia"[All Fields]) OR "pre eclampsia"[All Fields]) AND determinants[All Fields] AND ("ethiopia"[MeSH Terms] OR "ethiopia"[All Fields])=213
2. Hinari= 49: Pre-eclampsia and its determinants in Ethiopia.
