# Supplementary material for: Preeclampsia and its determinants in Ethiopia: A systematic review and meta-analysis
Source: PLoS One. 2023 Nov 14;18(11):e0287038. doi: 10.1371/journal.pone.0287038 (PMC10645334; doi:10.1371/journal.pone.0287038)
Supplement: S2 Table — (DOCX) [file pone.0287038.s003.docx]

**Additional file 2:** Newcastle-Ottawa Quality Assessment Scale for observational studies to assess the prevalence of preeclampsia among women in Ethiopia, 2023.

| Authors | Representatives | Sample size | Non -responders | Ascertainment | comparability | outcome | Quality score |
| --- | --- | --- | --- | --- | --- | --- | --- |
| Ayele et al (2021) | 1 | 2 | 1 | 1 | 1 | 1 | 7 |
| Birhanu et al (2020) | 1 | 2 | 1 | 2 | 1 | 1 | 8 |
| Tessema et al (2015) | 1 | 1 | 1 | 2 | 1 | 1 | 7 |
| Ayalew et al (2019) | 1 | 1 | 1 | 2 | 1 | 1 | 7 |
| Vata et al (2015) | 1 | 1 | 2 | 2 | 1 | 1 | 8 |
| Mareg et al (2020) | 1 | 2 | 1 | 1 | 1 | 1 | 7 |
| Demissie Beketie et al (2022) | 2 | 1 | 1 | 1 | 1 | 1 | 7 |
| Andarge RB et al (2020) | 1 | 1 | 1 | 1 | 2 | 1 | 7 |
| Belay & Wudad (2019) | 1 | 1 | 1 | 1 | 2 | 1 | 7 |
| Fikadu et al (2020) | 1 | 2 | 1 | 1 | 2 | 1 | 8 |
| Birhanu Jikamo et al. (2022) | 2 | 1 | 1 | 1 | 1 | 1 | 7 |
| Haile et al(2021) | 1 | 2 | 1 | 2 | 1 | 2 | 9 |
| Asres et al(2022) | 2 | 1 | 1 | 1 | 2 | 1 | 8 |
| Fantahunegne(2019) | 1 | 1 | 1 | 1 | 2 | 1 | 7 |
| Shegaze et al(2016) | 2 | 2 | 1 | 1 | 1 | 2 | 9 |
| Grum et al(2017) | 1 | 2 | 1 | 2 | 1 | 1 | 8 |
| Maereg Wagnew et al (2016) | 2 | 1 | 2 | 2 | 2 | 1 | 10 |
| Mohammed et al(2017) | 2 | 1 | 2 | 1 | 1 | 1 | 8 |
| Katore et al(2021) | 1 | 1 | 1 | 1 | 1 | 2 | 7 |
| Hinkosa et al(2020) | 1 | 2 | 2 | 1 | 1 | 1 | 8 |
| Legesse et al (2019) | 2 | 1 | 2 | 1 | 1 | 1 | 8 |
| Kahasy et al (2018) | 2 | 1 | 1 | 2 | 1 | 2 | 9 |
| Wodajo & Reddy (2016) | 1 | 2 | 2 | 1 | 2 | 2 | 10 |
| Tesfaye et al(2019) | 2 | 1 | 2 | 1 | 1 | 1 | 8 |
| Gudeta et al(2018) | 2 | 1 | 1 | 2 | 1 | 2 | 9 |
| Terefe et al(2015) | 1 | 2 | 2 | 1 | 1 | 2 | 9 |
| Asfaw (2014) | 1 | 2 | 1 | 2 | 1 | 1 | 8 |
| Mekie et al(2020) | 2 | 1 | 2 | 2 | 2 | 1 | 10 |
| Yitagesu Belayhun et al(2021) | 2 | 1 | 2 | 1 | 1 | 1 | 8 |
| Alemie(2021) | 1 | 1 | 1 | 1 | 1 | 2 | 7 |

**Interpretation of the score**: Very Good Studies: 9-10 points, Good Studies: 7-8 points, Satisfactory Studies: 5-6 points and Unsatisfactory Studies: 0 to 4 points
