# Supplementary material for: Preeclampsia and its determinants in Ethiopia: A systematic review and meta-analysis
Source: PLoS One. 2023 Nov 14;18(11):e0287038. doi: 10.1371/journal.pone.0287038 (PMC10645334; doi:10.1371/journal.pone.0287038)
Supplement: Attachment — Submitted filename: Response to Reviewers.docx [file pone.0287038.s004.docx]

**A point-by-point response to the reviewers**

When submitting your revision, we need you to address these additional requirements.

Please ensure that your manuscript meets PLOS ONE's style requirements, including those for file naming. The PLOS ONE style templates can be found at https://journals.plos.org/plosone/s/file?id=wjVg/PLOSOne_formatting_sample_main_body.pdf and <https://journals.plos.org/plosone/s/file?id=ba62/PLOSOne_formatting_sample_title_authors_affiliations.pdf>

Response: we accept the comment and tried to correct based on the journal submission guideline.

Additional Editor Comments:

Given that there are similar systematic reviews and meta-analyses conducted on this topic, can the authors please justify the need for this study to be conducted? What is the novelty of their research? How it is different from prior meta-analyses and what is it significantly adding to the current literature? Also, please add the clinical implications of your findings.

Response: we accept your comment, but the previous systematic review and meta-analysis were conducted generally in the hypertensive disorder of pregnancy before three and five years respectively, while this study only focuses only on preeclampsia including the recently published articles in order to find the exact recent figures. It is the first study in Ethiopia and it shows preeclampsia is still prevalent. The clinical implication of this study is the prevalence of preeclampsia is still high; the clinician should be focused on prevention strategies for preeclampsia. In addition, during patient diagnosis and management, clinicians will conduct detailed patient evaluations to identify the risk factors of pre-eclampsia and to develop a better treatment protocol. For more information see the conclusion part.

Reviewer #1: Authors conducted a meta-analysis to determine the prevalence and determinants of preeclampsia in Ethiopia. Manuscript is well written; tables and figures are well made. I have few comments to improve the current version.

Abstract

1. Please replace “therefore” or “hence” with “as a result” in the background section.

Response: we accept your comment and updated the main document based on the given direction.
2. The following sentences can be merged for sake of conciseness and clarity.
“A systematic review and meta-analysis were conducted on observational facility-based studies between the years January 1, 2013, and January 1, 2023, in Ethiopia. Electronic searches such PubMed, Google Scholar, HINAR, Scopus, Web of Sciences and grey literature were used to 37 search primary studies.”

Response: we accept your comment and amend the document accordingly.
3. Use of the word “such as” gives an impression as if not all the databases that were searched to identify articles are mentioned. If that is the case, please mention all the databases in methods or eliminate the phrase.

Response: we accept your comment and amend the document accordingly.
4. Please mention the name of “Standardized measurement tool” that was used to extract data.

Response: we accept the comment, and this standardized measurement tool is a Microsoft Excel forms consisting of the author name, year of publication, study setting, sample size, study design, population, response rate, prevalence of preeclampsia and factors of preeclampsia.
5. “STATA 11 statistical software was used to analyze the data as well and I2 tests evaluated study heterogeneity”
Please delete as well from this sentence.

Response: we accept the comment and amend the document accordingly.
6. Were there p-values along with AOR? They maybe worth mentioning in the results.

Response: we accept the comment, but did not necessarily to put p-value once mention the adjusted odd ratio; which is more informative.
7. Conclusion is too big for abstract please try stating conclusions in maximum of 2-3 short sentences. This conclusion is too big for whole manuscript as well. Consider rephrasing.

Response: we accept the comment and updated based on your direction.
Introduction
1. It might be worth mentioning exact figures of worldwide prevalence of preeclampsia.

Response: we accept your comment and recommendation, and the prevalence of preeclampsia difference across each counties with different risk factors; we tried to put the figure in the main document.
2. Current version of introduction is very long and provides very general details regarding preeclampsia. Please shorten it and focus more towards building rationale of this particular study.

Response: we accept the comment and recommendation and amend the introduction accordingly.

Methods
1. All studies were published in Ethiopia? Or all studies were conducted in Ethiopia?

Response: all studies were conducted in Ethiopia.
2. Please state the criteria for including studies in a paragraph form.

Response: we accept the comment and recommendation and updated based on the given information.
3. Why were studies not taken from inception and taken from January 2013?

Response:

- In recent; healthcare facilities, skilled birth attendants, and diagnostic modalities are better to identify preeclampsia. Due to this situation, we use the recent ten years of articles.
- Scientifically recommended that we use the recent ten-year published article to generalize the findings of the study, based on this we are starting from 2013.
- As the healthcare infrastructure improved, service also improved leading to reporting such types of cases, and better representing the recent ten years than before.

Results
1. Results are well described. Please mention the t value for egger’s test if it is available.

Response: we accept the comment and recommendation, and t value for Egger’s test= 8.30, p > │t│=0.000
2. Replace Magnitude of Preeclampsia with Magnitude of Preeclampsia Prevalence or equivalent.

Response: We accept the comment and recommendation, and update based on the given direction
3. Semi-colon is not correctly used. Please correct grammar throughout.

Response:
4. Please mention p-values alongside AOR for determinants, if available.

Response: We accept the comment and recommendation and tried to update the main document based on the given direction.
Conclusion
1. Please shorten the conclusion. Currently, it provides a lot of redundant details. It should be one single paragraph, providing all the takeaway points.

Response: we accept the comment and recommendation and corrected accordingly.

Reviewer #2: 1. Please structure the Methods section under the headings of Search strategy, Study selection criteria, Screening process and data extraction, Extracted parameters, Quality assessment (ROB), and Statistical Analysis.

Response: we accept your comment and recommendation and we tried to correct accordingly.
2. Please indicate how the screening of articles was done in more detail such as which reviewers performed the screening.

Response: Accept the comment and done based on your given direction.
3. Kindly explain the rationale behind “Variables were chosen for this study if they were reported as a significant factor in two or more studies.”

Response: we accept your comment and this sentence message is in order to compute the odd ratio of the pooled estimate the included studies at least have one common associated factor.

4. In line 130, please make the phrase “up to December January 1, 2023” clearer.

Response: we accept the comment and corrected it accordingly.
5. The part concerning operational definitions should be mentioned under the heading of “Extracted parameters”.

Response: we accept the comment and amend it accordingly.
6. There are grammatical errors in various places throughout the text, please correct them.

Response: accept and we tried to rephrase and rewrite by using different online software.
7. State the full forms of all abbreviations the first time they are used in the text.

Response: Accept the comment and done based on your given direction.
8. Please be precise while wording the results section.

Response: we accept your comment and recommendation and we tried to correct them accordingly.
9. Kindly improve the language of the Results section, while correcting for any grammatical and spelling mistakes. For example, in Lines 216 - 222.

Response: we accept the comment and amended it accordingly.
10. Edits to be made to Line 62 – pregnancy-induced hypertension and Line 72 – multiparous.

Response: We accept the comment and amended accordingly.
11. Kindly shorten the Introduction by avoiding repetition and by making it more concise.

Response: We reviewed based on your comment and corrected/ amended it accordingly.
12. Please remove/replace all references older than 10 years.

Response: we accept the comment and tried to amend the paper based on the given direction.
