# Supplementary material for: Preeclampsia and its determinants in Ethiopia: A systematic review and meta-analysis
Source: PLoS One. 2023 Nov 14;18(11):e0287038. doi: 10.1371/journal.pone.0287038 (PMC10645334; doi:10.1371/journal.pone.0287038)
Supplement: Attachment — Submitted filename: Response to reviewer.docx [file pone.0287038.s005.docx]

**Point by point response to the editor and authors**

Journal Requirements:

Please review your reference list to ensure that it is complete and correct. If you have cited papers that have been retracted, please include the rationale for doing so in the manuscript text, or remove these references and replace them with relevant current references. Any changes to the reference list should be mentioned in the rebuttal letter that accompanies your revised manuscript. If you need to cite a retracted article, indicate the article’s retracted status in the References list and also include a citation and full reference for the retraction notice.

Response: we accept the comment and there is no used retracted article in the reference list.

Additional Editor Comments:

The authors have responded to the query regarding clinical implications of this article. However, no significant text pertaining to implications has been added to the main text of the manuscript. Kindly add clinical implication details to the discussion section of the manuscript wherever the authors feel it is appropriate. Please ensure that the flow and coherence of the discussion text is maintained.

Response: we accept the comment and we tried to update the main document accordingly.

**Reviewer #1:** All of my comments have been addressed except for few

1. Request to mention p-values alongside AOR throughout the manuscript. Authors mention that p-values are not required with AOR, which is not correct. P-value is absolutely needed to determine whether the stated association is statistically significant or just by chance and hence ignorable. Therefore, it is advised to please mention p-values throughout.

Response: we accept the comment, and update based on the given information.

2. In the abstract, please explicitly mention you used Microsoft Excel. "Standardized data collection tool" is not sufficient information.

Response: we accept the comment and amend based on the given direction.

3. In the methods section, please specifically mention the reason you included the article between 2013 and 2023.

Response: based on the following reason we restricted data extraction from 2013;

- In recent; healthcare facilities, skilled birth attendants, and diagnostic modalities are better to identify preeclampsia. Due to this situation, we use the recent ten years of articles to generate the evidence about pre-eclampsia.
- Scientifically recommended that we use the recent ten-year published article to generalize the findings of the clinical the study, based on this we are starting from 2013.
- As the healthcare infrastructure improved, service also improved leading to reporting such types of cases, and better representing the recent ten years than before.

**Reviewer #2:** The authors have improved upon the quality of their article. I have some minor comments to make, after which I believe the article would merit publication. These comments are as follows;

1. I believe the Introduction section can still be restructured in a better manner.

Response: we accept the comment and recommendation and amend the document accordingly

2. Line 75-76: Rephrase as "Global studies reveal that the incidence of pre-eclampsia in nulliparous females ranged from 3 to 10% while in multiparous women, the incidence of preeclampsia ranged from 2 to 5%"

Response: We accept the comment and corrected based on the given information.

3. Line 91 - It should just be stated "Preeclampsia and eclampsia are associated with hypertension and are known to poorly impact maternal and newborn mortality and morbidity"

Response: We accept the comment and amend based on the given information

4. In Lines 93-95, the first sentence and the phrase, "increases caesarian delivery rate", need to be rephrased.

Response: We accept the comment and tried to rephrase it accordingly

5. Line 99 - The term "hypertension problems in women" needs to be better phrased.

Response: we accept the comment and rephrased based on the given direction.

6. Line 109 - Is it "across each region" or across various regions in the state?

Response: amended as ‘across various regions in the state.’

7. Line 132 - Replace "whereas" with meanwhile.

Response: replaced with the recommended one

8. Line 166 - Rephrase "through a two investigator (BCD and SA)"

Response: we accept the comment and rephrased based on the given direction.

9. Line 168 - kindly mention that it is an MS Excel sheet that includes (not "considers") data concerning the variables stated in the text.

Response: Response: we accept the comment and update the document based on the given direction.

10. Line 199 - Please replace "done to find" with "performed to ascertain"

Response: corrected based on the given direction

11. Line 205 - Please edit it as "Of these articles, 972 duplicates were eliminated while 1536 articles were reviewed further for inclusion"

Response: We accept the comment and replaced it

12. Line 216 - Specify Figure or Table along with S2. Additionally, exclude "see" from all brackets in the entire text where you are referring to a table or figure.

Response: done it based on the given direction

13. Line 220 - It should be "reported" instead of "report"

Response: changed based on your recommendation

14. Line 225 - Use indicating instead of "showing"

Response: updated the document based on your recommendation.

15. Line 227 - Incorporate the t value for Egger's regression test.

Response: we accept the comment and add the t-value in the document based on the given direction.
